# Impact of Preoperative Immunonutrition on Oxidative Stress and Gut Barrier Function in Surgical Patients with Crohn’s Disease

**DOI:** 10.3390/nu15040882

**Published:** 2023-02-09

**Authors:** Elisabetta Bigagli, Mario D’Ambrosio, Lorenzo Cinci, Camilla Fiorindi, Sara Agostiniani, Elisa Bruscoli, Anita Nannoni, Maura Lodovici, Stefano Scaringi, Francesco Giudici, Cristina Luceri

**Affiliations:** 1 Department of Neurosciences, Psychology, Drug Research and Child Health (NEUROFARBA), University of Firenze, 50134 Firenze, Italy; 2 Department of Health Science, University of Firenze, 50134 Firenze, Italy; 3 Department of Experimental and Clinical Medicine, University of Firenze, 50134 Firenze, Italy

**Keywords:** Crohn’s disease, AGEs, AOPP, RAGE, oxidative stress, immunonutrition, barrier function

## Abstract

Several international guidelines recommend a peri-operative immunonutrition (IN) support for patients care in elective colorectal surgery, to reduce postoperative complications, particularly infections. In Crohn’s patients, is also used to mitigate the severity of the disease. We performed a pilot study on 16 Crohn’s patients undergoing intestinal surgery for active disease, not responsive to pharmacological treatment; half of them received an oral nutritional supplement enriched with immunonutrients (IN patients) for 7 days prior to surgery, in addition to normal food intake. Markers of oxidative stress (Advanced Glycated End-products (AGEs) and Advanced Oxidation Protein Products (AOPPs) were measured both in plasma and tissue samples wherein the Receptor for Advanced Glycation End products (RAGE) and Tight Junction Protein 1 (TJP1) gene expression were also determined. Plasma AGEs were significantly and positively correlated with tissue levels of AGEs (*p* = 0.0354) and AOPPs (*p* = 0.0043) while they were negatively correlated with TJP1 expression (*p* = 0.0159). The expression of RAGE was also negatively correlated with that of TJP1 gene (*p* = 0.0146). IN patients exhibited significantly lower AGEs plasma levels (*p* = 0.0321) and a higher mucosal TJP1 expression (*p* = 0.0182). No patient had postoperative complications and the length of hospital stay was similar in the two groups, but IN patients, showed a significantly shorter time to resume fluid and solid diet. These preliminary data suggest that IN might support patient’s recovery by improving intestinal mucosa barrier function through the regulation of AGEs/RAGE signaling.

## 1. Introduction

Crohn’s Disease (CD) patients are often affected by various grades of malnutrition because of impaired absorption and loss of nutrients, secondary to severe diarrhea and intestinal fistulae [1].

Enteral nutrition (EN) has been studied in CD for decades, in particular in the pediatric population, proving to be as effective in inducing remission as steroids [2]. Beneficial effects ascribed to EN include the reduction in disease activity scores, mucosal healing, and downregulation of mucosal pro-inflammatory cytokines with mechanisms including relative bowel rest, reduced antigenic load, provision of trophic amino acids, local anti-inflammatory effects, and modification of bowel microflora [3,4].

Recently, immunonutrition (IN) formulas have been added to the standard nutritional interventions, supplemented with biologically active nutrients such as arginine, omega-3 fatty acids, glutamine, and RNA, among others [5]. The rationale of adding immunonutrients to standard nutritional support is to boost the systemic and gut immune response improving the control of inflammation and tissue regeneration.

Nutritional status is a key factor influencing clinical outcomes and nutritional supports are also often used in patients undergoing major intestinal surgery. In the last years, IN has gained increasing interest, it is now recommended in several international guidelines and has been included in the Enhanced Recovery After Surgery (ERAS) protocol for colorectal surgery [6].

In surgical IBD patients, inadequate body composition and malnutrition are associated to a higher risk of postoperative complications and to a longer hospital stay [7]. Thus, IN has the potential to improve surgical outcomes also in IBD patients, by reducing the incidence of postoperative complications and shortening the hospital stay. Although IN has been used in clinics for over 20 years, results are not always consistent and there is debate as to whether it should also be used in well-nourished patients [8]. However, since surgical injury may alter the mucosal structure, potentially resulting in a loss of the gut barrier function, IN may be beneficial regardless of nutritional status [9].

In CD patients, the hyperactivation of the immune system and chronic inflammation promote the production of reactive oxygen species (ROS) thereby increasing oxidative damage, however, several evidence suggests that oxidative stress itself may be a disease-triggering factor and a key effector of mechanisms leading to tissue injury [10]. ROS are involved in several intracellular signaling regulating cell growth, differentiation, death, and inflammation. Indeed, redox signaling are involved in the upregulation of pro-inflammatory cytokines and in the increased infiltration of inflammatory cells in particular via stimulating the redox-sensitive transcription factor nuclear factor kappa B (NF-kB) [11].

Increased levels of circulating Advanced Oxidation Protein Products (AOPPs) and of Advanced Glycated End-products (AGEs) have been reported in the serum/plasma of CD patients [12,13]. AOPP and AGEs are both ligands of the Receptor for Advanced Glycation End-products (RAGE), a transmembrane, multi-ligand receptor, expressed by a wide range of cells and upregulated in chronic diseases, including CD, mainly in inflamed areas [14].

In experimental models, it has been reported that AOPPs reduced intestinal mucosa integrity [15], and AGEs reduced the expressions of the tight junction proteins Zonula occludens-1 (ZO-1), also known as Tight junction protein-1 (TJP1), and Occludin, by activating RAGE/NF-kB pathway suggesting that RAGE signaling down-regulates proteins involved in barrier function [16].

We herein reported the results of a pilot study on a small cohort of CD patients with complicated disease requiring surgery. Our aim was to evaluate the effects of a preoperative IN on circulating and mucosal levels of AOPP and AGEs and its possible impact on post-surgical outcomes. While at circulating level we did not observe differences between groups, locally, IN significantly reduced the amount of AGEs and increased the expression of TJP1. Moreover, patients who underwent a perioperative IN protocol resumed liquid and solid diet earlier compared to controls suggesting positive effects also on post-surgical recovery.

## 2. Materials and Methods

### 2.1. Patient’s Enrollment and Sample Collections

This study was approved by the Research Ethics Committee of the Hospital of Careggi, Florence, Italy (12382_BIO, 11/6/2018). After obtaining written informed consent, 16 CD patients were recruited between September 2019 and January 2020, at the Digestive Surgery Unit of Careggi Hospital. The patients enrolled in the study underwent surgery for active disease, not responsive to pharmacological treatment and/or with a complicated disease. Patients were matched for age, gender, disease location and smoking habit.

Mucosa and blood samples were collected at surgery; blood samples were collected in EDTA coated tubes, centrifuged at 2000 rpm for 10 min to isolate plasma, and stored at −20 °C, like tissue samples, until analysis.

Demographic and clinical data (age, gender, body mass index, smoking habit, familial IBD, disease duration, disease location, the interval between diagnosis and surgery, number of surgeries, presences of abdominal fistulae, disease behavior, type of surgery, therapies before surgery, short-term post-operative complications and length of hospital stay) were retrieved from medical records. Disease activity was defined according to the CD activity index (CDAI).

A dedicated dietitian of our Inflammatory Bowel Disease (IBD) Unit evaluated the nutritional status of each patient during the pre-hospitalization assessment; the physicians involved in the study were blinded regarding any nutritional intervention. All patients were screened for malnutrition with the Nutritional Risk Screening (NRS-2002) tool [17]. Patients with a score ≥3 were considered nutritionally at-risk.

Half the patients enrolled were asked to consume 2 cartons (400 mL) of Impact^®^ Oral (Nestlè) per day, for 7 days prior to surgery, in addition to normal food intake, regardless of their nutritional status (IN group); in the control group, malnourished patients received standard nutritional support, without IN. No patients were so malnourished as to require enteral nutrition.

### 2.2. Markers of Oxidative Stress

AOPPs were measured in plasma and tissue samples. For plasma determination, 20 μL of plasma and 980 μL of PBS were mixed to 50 μL of KI 1.16 M and 100 μL of acetic acid.

The absorbance of the reaction mixture was read at 340 nm and the AOPPs were quantified in μmol/mg of proteins using Chloramine-T (Sigma-Aldrich, Milan, Italy) as standard for the calibration curve.

AGEs were determined on 100 μL of 1:5 PBS-diluted plasma, reading the fluorescence intensity at 460 nm, after excitation at 355 nm. Results were expressed as arbitrary units (AU).

Tissues were first homogenized using an Ultraturrax homogenizer on PBS and subsequently centrifuged at 13,000 rpm at 4 °C. The supernatant (400 µL) was divided into aliquots used for AOPPs and AGEs determination as described above. Proteins content in plasma and tissue samples was determined by using the Bio-Rad DC protein assay kit (Bio-Rad, Milan, Italy) and albumin as standard for the calibration curve.

### 2.3. RT-PCR

The Nucleo Spin^®^ RNA kit (Macherey-Nagel, Bethlehem, PA, USA) was used to extract total RNA from tissue homogenates.

For first-strand cDNA synthesis, about 100 ng of total RNA from each sample were reverse-transcribed by using the RevertAid RT Kit (ThermoScientific, Waltham, MA, USA) according to the manufacturers’ protocol.

Primers were designed based on the GenBank sequences for Homo sapiens Tight Junction Protein 1, TJP1 (NM_003257.4): forward primer 5′-GGGAGCACATGGTGAAGGTAA-3′, reverse primer 5′-ATCACAGTGTGGTAAGCGCA-3′ and of Homo sapiens Advanced Glycosylation End-Product Specific Receptor, RAGE (NM_001136.5): forward primer 5′-GGAAAGGAGACCAAGTCCAA-3′, reverse primer 5′-CATCCAAGTGCCAGCTAAGA-3′. GAPDH was co-amplified as reference gene: forward primer 5′-CCCTCAAGGGCATCCTGGGCT-3′, reverse primer 5′-GCAGGGACTCCCCAGCAGTGA-3′. PCRs were carried out using 1 μL of cDNA in a 25μL total volume containing 1 × PCR buffer, 0.5 mM dNTPs, 8 ng/μL of primer, 0.1 ng/μL of GAPDH primers and 1.25 units of Taq polymerase (Dream Taq, Carlo Erba, Milan, Italy). The PCR conditions were: 95 °C for 5 min and 30 cycles at 95 °C for 30 s, 60 °C for 30 s and 72 °C for 55 s. PCR products were separated on an agarose gel (1.8%) and visualized by Safeview staining (Euroclone, Milan, Italy). Gel images were captured by an UVIdocHD2 acquired system (Eppendorf, Milan, Italy) and the intensity of the bands were analyzed with the Quantity-One software (Bio-Rad, Segrate, Milan, Italy).

### 2.4. Statistical Analyses

Statistical analyses were performed using Graph-Pad Prism 8.01 and Statgraphics Centurion XVI software. Normality was verified with the Kolmogorov-Smirnov test and normally distributed data were expressed as means ± standard error (SE) and analyzed by Student’s *t*-test. Non-normally distributed variables, expressed as median and interquartile range, were analyzed by unpaired *t* test. Differences between proportions were assessed using the chi-square or Fisher exact test. Correlations among continuous variables were analyzed by linear and multiple regression. A stepwise multiple linear regression analysis was performed with oxidative stress markers as dependent variables and the following factors as independent variables: age at surgery, gender, smoke habit, surgical recurrence, CDAI, disease duration and nutritional risk. *p*-values less than 0.05 were considered statistically significant.

## 3. Results

The demographics and clinical characteristics of CD patients included in this study were similar. No significant differences were observed in age, gender, BMI, smoking habit, disease duration, disease behavior and location, between IN and control patients (Table 1). All patients underwent an ileocecal resection that in most of the cases was performed by a minimally invasive surgical approach (laparoscopy). One patient in the IN group and one patient in the control group underwent an open surgery.

Regarding pre-operative medications, there were no differences in the number of patients treated with 5-aminosalicylate (5-ASA) or corticosteroids between the two groups. In any case, pharmacological therapies, if any, were suspended at least two weeks before surgery.

CD patients in the IN group had a slightly higher CDAI compared to the control group, but this difference did not reach statistical significance (*p* = 0.1189) (Table 1).

The nutritional status of CD patients in the IN group was similar to those in the control group; 62.5% of controls and 50% of IN patients had a score >3 according to the NRS-2002 screening system that takes into account the undernutrition (BMI, percent of recent weight loss and change in food intake) and disease severity (absent, mild, moderate or severe) of the patient.

**Figure 1:**
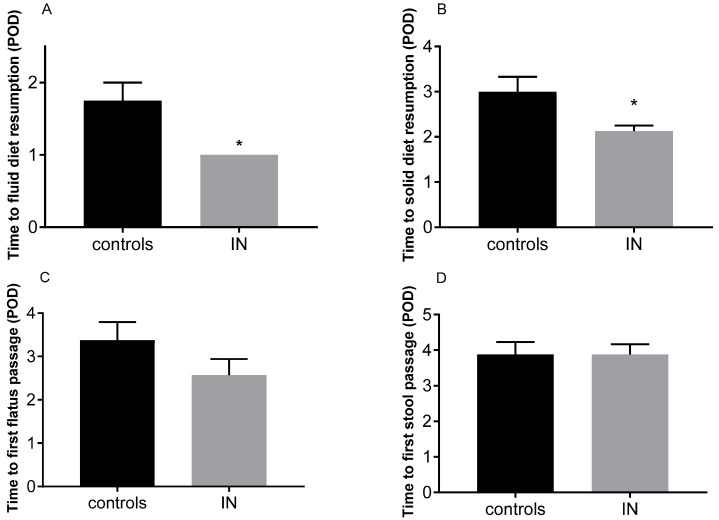
Mean days of postoperative (POD) fluid (**A**) and solid oral diet resumption (**B**) and time of first bowel movement, considering both the first POD flatus passage (**C**) and stool passage (**D**) in controls and in IN patients. Data are expressed as means ± SEM * *p* < 0.05 by unpaired *t* test.

**Figure 2:**
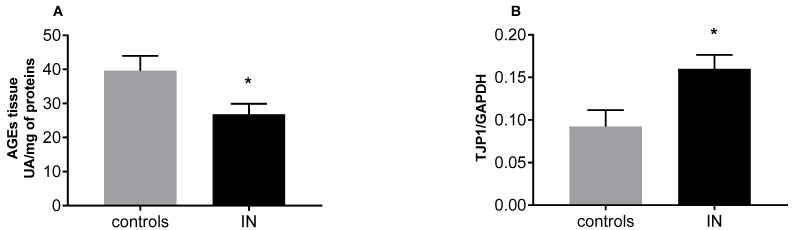
Mean levels of Advanced Glycated End-products, AGEs (**A**) and of Tight junction protein-1 (TJP1) gene expression (**B**) in mucosal samples from CD patients in the control and IN groups. * *p* < 0.05 by unpaired *t* test. UA = Arbitrary Unit.

On the contrary, among post-operative outcomes, we found that IN patients had a faster oral nutrition resumption. Indeed, in the IN-group, all patients resumed oral fluid nutrition at postoperative day (POD) 1 compared to the 37.5% of control patients (*p* = 0.0256, Table 2 and Figure 1, panel A). Moreover, 87.5% of the IN patients resumed solid food intake at POD2 compared to the 25% of patients in the control group (*p* = 0.0286, Table 2 and Figure 2, panel B). No significant differences were observed in time to first POD flatus passage or time to first POD stool passage between the IN and control groups (Figure 1, panels C and D).

While we did not observe differences in the plasma levels of both AGEs and AOPPs comparing the two groups, the amount of AGEs in tissue samples was significantly lower in the IN-group compared to controls (26.83 ± 3.06 vs. 39.59 ± 4.41 UA/mg of proteins; *p* = 0.0321) (Table 3 and Figure 2, panel A).

Interestingly, TJP1 gene expression in mucosal samples from CD patients in the IN group was significantly higher than in controls (Figure 2, panel B), while RAGE expression was similar (0.88 ± 0.1 vs. 0.89 ± 0.15 relative expression).

The levels of AGEs in tissue samples were significantly lower in IN patients even after adjustment for demographics (age, gender, and smoke habit) or clinical factors (surgical recurrence, CDAI, disease duration and nutritional risk), data not shown.

**Figure 3:**
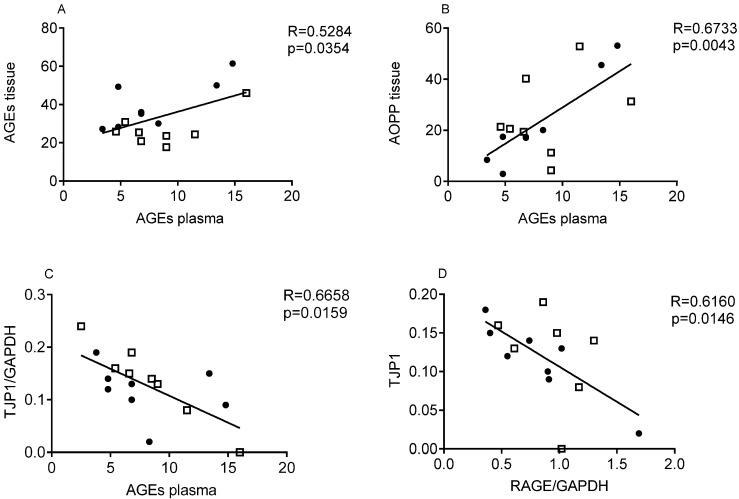
Correlation between Advanced Glycated End-products (AGEs) levels in the plasma of CD patients and in mucosal samples (**A**); correlation between AGEs levels in the plasma and Advanced Oxidation Protein Products (AOPPs) in mucosal samples (**B**); correlation between AGEs levels in the plasma and Tight junction protein-1 (TJP1) gene expression (**C**) in mucosal samples; correlation between TJP1 and Receptor for Advanced Glycation End products (RAGE) gene expression (**D**) in mucosal samples. Dotted symbols (●) represent controls; square symbols (⎕) represent IN patients.

Circulating AGEs were positively and significantly correlated to their tissue levels (Figure 3, panel A, *p* = 0.0354), to AOPP plasma levels (Figure 3, panel B, *p* = 0.0043) and to TJP1 gene expression, in mucosal specimens (Figure 3, panel C, *p* = 0.0159). Interestingly, we also found a negative correlation between TJP1 and RAGE gene expression (Figure 3, panel D, *p* = 0.0148).

## 4. Discussion

Malnutrition is a frequent occurrence in CD patients and a systematic review on preoperative nutritional support in adult CD over 20 years, indicated that malnutrition is a major risk factor for postoperative complications and that preoperative nutritional support decreases morbidity after surgery [18].

A perioperative IN is recommended based on a high level of evidence in the setting of gastrointestinal cancer surgery [8,19]: it should be started after nutritional screening and for a minimum of five days, depending on the degree of malnutrition and the type of surgery [20]. In this way, IN seems to reduce postoperative complications, particularly infections, along with the length of hospital stay [8,21]. Moreover, providing immunonutrition could have a beneficial effect for both well-nourished and poorly nourished patients since in both cases, surgical stress may alter the mucosal barrier structure and function and gut inflammation.

So far, the molecular mechanisms mediating the beneficial effects of IN have been scarcely investigated.

In this study, preoperative IN was used in CD patients regardless of their nutritional status, and its effects on post-operative outcomes were investigated along with molecular insights on its effects in the intestinal mucosa and in circulating and mucosal markers of oxidative damage.

Despite the small number of patients enrolled, the two groups were well matched, regarding their demographic, clinical and surgical data. All our patients required surgery due to an acute or complicated disease, refractory to pharmacological therapy; 37.5% of the patients in both groups were treated with corticosteroids and similarly no differences were found regarding pre-operative use of ASA. In anyway, all therapies were suspended at least two weeks before surgery.

The percentage of patients at nutritional risk was similar between groups (50% in IN and 62.5% in the control group). The number of patients with surgical recurrence was higher in the control group (71.4% vs. 28.6%) while the percentage of patients with abdominal fistulae (62.5% vs. 25%) and the CDAI values (300, 250–350 vs. 250, (250–300; median, IQ) were higher in IN patients but none of these differences reached statistical significance. Most of the patients (14 out of 16; 87.5%) underwent an ileocecal resection by a minimally invasive surgical approach and no patient experienced postoperative complications. The length of hospital stay was similar in the two groups, however, IN patients showed a significantly shorter time to resume the fluid and solid diet compared to the control group.

The mean plasma levels of AOPP and AGEs were similar between groups and the AOPP concentrations were comparable in mucosal samples. On the contrary, we observed a significant reduction in the amount of AGEs in the inflamed mucosa.

AGEs are a class of heterogeneous compounds derived from proteins, lipids, and nucleic acids, non-enzymatically glycated by reducing sugars, produced endogenously, in highly processed food and in cigarette smoke [22]. An increased amount of AGEs is observed in the presence of hyperglycemia and oxidative stress [23,24].

AOPPs are biomarkers widely used to estimate both circulating and tissue protein oxidative damage; AOPPs result from the reaction of proteins with chlorinated oxidants such as hypochlorous acid via the action of neutrophil myeloperoxidase [13].

We also recently demonstrated that AGEs and AOPPs are increased in the blood of CD patients with severe disease compared to controls [13]. AGEs may affect membrane integrity both through the glycation of tight junction proteins and by activating RAGE signaling [22].

The activation of RAGE by its ligands, initiates complex signaling pathways, including the activation of NADPH oxidase, p21ras GTPase, ERK1/2 and MAP kinases and the JAK/STAT pathway, with downstream inflammatory consequences, including the activation of NF-kB. Notably, NF-kB itself modulates the expression of RAGE, maintaining and boosting the signal [25]. Moreover, RAGE is involved in a crosstalk with Toll-like receptors to coordinate and regulate immune and inflammatory responses [26].

Recently Body-Malapel and coworkers reported that mice lacking RAGE are less susceptible to intestinal inflammation and that WT mice treated with a RAGE-specific inhibitor were protect from indomethacin-induced enteritis and DSS-induced colitis [27]. The role of the AGE-RAGE axis as modulator of gut permeability is well described in diabetes [28], but emerging evidence supports its role also in other contexts. AGEs increased placental vascular permeability of human BeWo cells reducing the expressions of the transmembrane proteins ZO-1 and Occludin and an anti-RAGE antibody or a NF-kB inhibitor, partially restored their levels [16]. In rats fed an AGEs diet, the expressions of Occludin and ZO-1 were reduced and the structure of colonocytes altered, demonstrating that the epithelial barrier was partially damaged [29].

Accordingly, we observed that the expression of TJP1, also known as ZO-1, was significantly higher in IN patients who also exhibited a lower AGEs level; moreover, TJP1 expression was negatively correlated with that of RAGE.

Impact^®^ Oral contains components that may interfere with the immune system: glutamine, arginine, omega-3 fatty acids and nucleotides.

Glutamine is one of the precursors of glutathione synthesis and serum L-glutamine levels in CD patients are lower than in healthy individuals [30]. A number of experimental studies in rodents demonstrated that L-glutamine alleviates IBD, administered before, after or during the induction of colitis [31]. L-glutamine exhibited antioxidant and anti-inflammatory effects andattenuated endoplasmic reticulum stress and apoptosis [32]. In intestinal porcine epithelial cells and in the jejunum of weanling piglets, L-glutamine enhanced the barrier integrity and function by increasing the expression of TJ proteins ZO, occludin, claudins, and JAM-A [33,34].

In addition to being a building block for several cellular proteins such as ornithine, proline, glutamic acid, glutamine and creatine, L-arginine plays a critical role in enhancing the immune system, wound healing and tissue regeneration: dietary supplementation of L-arginine protected against colitis by modulating gut microbiota [35], and reduced intestinal permeability, markers of oxidative stress and inflammation [31]. Furthermore, L-arginine can regulate many signaling pathways, being a substrate of nitric oxide (NO) synthases thus allowing the generation of physiological NO amounts. It is also a precursor of the formation of polyamines, which are important for connective tissue repair [36].

It has been suggested that L-arginine mays unveil its full potential only if combined with other micronutrients. In active CD patients, the supplementation of arginine alone, did not increase NO production while in combination with glutamine, reduced the release of pro-inflammatory cytokines [37]. A systematic review on the perioperative use of arginine supplementation evidenced that the rate of infectious complications was reduced with Impact^®^ Oral and not with other arginine formulations [38].

There is no consensus on the efficacy of n-3 polyunsaturated fatty acids (n-3 PUFA) in the prevention or treatment of IBD; the latest Cochrane review concluded that they are probably ineffective for maintenance of remission in CD [39], and they are currently not recommended by the ESPEN 2017 guidelines for the treatment of CD patients [40]. However, several data demonstrated their effects in reducing inflammation, probably in virtue of their ability to change cell membrane composition and to activate the peroxisome proliferator activated receptor γ. Moreover, some studies showed that n-3 PUFA reduced intestinal permeability and improved intestinal morphology and barrier function [41]. In Caco2 cells, docosahexaenoic acid (DHA) limited the effect of the inflammatory stimulus on occludin, ZO-1 and barrier function [42], inhibited the NF-kB pathway and ameliorated experimental colitis in IL-10-/- mice, improving intestinal epithelial barrier function [43].

As components of the nucleic acids DNA and RNA, nucleotides are integral components of almost all physiological processes and in conditions characterized by an increased demand for nucleic acid synthesis, including gut injury, exogenous supplies may be beneficial to cells undergoing rapid turnover such as those of the gut and immune systems.

The postulated influence of nucleotides in IN is in fact related to their relative deficiency during severe inflammation secondary to their excessive use by immune cells. Dietary nucleotide supplementation showed beneficial effects on the gut under stressful conditions, improved intestinal recovery, increased adaptive growth of the small intestine, and increased absorption capacity under malabsorption conditions [44]. Regarding surgical stress, nucleotides have been shown to play a role in immune cell synthesis, tissue repair, and in maintaining organ function [45].

There are few data on the perioperative use of IN in CD patients. The systematic review by Grass and coworkers (2017) identified 14 studies, mainly retrospective, half with exclusive enteral nutrition (EEN) and/or half with total parenteral nutrition [18]. There were only two studies reporting the use, both as EEN, of TGF-β-based IN: Heerasing et al. (2017) reported that patients pre-optimized with Modulen-IBD had significantly fewer surgical complications than control patients [46]. On the contrary, Beaupel and colleagues (2016), using an exclusive polymeric diet enriched with TGF-β in high-risk patients, did not find difference in the rate of postoperative complications between these cases and low-risk patients [47].

In patients undergoing elective surgery, Impact^®^ Oral supplementation was associated with significant reductions in postoperative infectious complications and with a significant decrease in hospital stay. Moreover, the greatest improvement in postoperative outcomes was observed in patients receiving preoperative nutritional support [48].

While aware that this is a small–size pilot study, our results suggest that IN might beneficially modulate mechanisms that promote local intestinal inflammation and compromise mucosal integrity and that its effects should be explored in a specifically designed clinical trial. Moreover, this data suggest that IN may enhance mucosal integrity and reconstitution by down-regulating the AGE/RAGE axis and increasing TJP1 expression.

## Figures and Tables

**Table 1:** Demographics and clinical characteristics of the two groups of Crohn’s disease patients.

	Controls	Immunonutrition	*p*
***n***	8	8	
**Age**, years	49.0 ± 5.1	50.5 ± 4.3	0.8270
**Gender M**, n (%)	5 (62.5%)	4 (50.0%)	>0.999
**Smokers/former smoker**, n (%)	5 (62.5%)	4 (50.0%)	>0.999
**BMI**, kg/m2	23.1 ± 1.43	20.92 ± 1.1	0.2484
**Disease duration**, yrs	18.2 ± 3.2	12.3 ± 3.5	0.2274
**Familial IBD**, n (%)	0	2 (25.0%)	0.4667
**Disease location ileal**, n (%)	8 (100%)	8 (100%)	>0.999
**Disease behavior**			>0.999
Stricturing	4	4	
Fistulizing	4	4	
**Nutritional risk**, n(%)			>0.999
1–2	3 (37.5%)	4 (50.0%)	
3–4	5 (62.5%)	4 (50.0%)	
**Surgical recurrence** n (%)	5 (71.4%)	2 (28.6%)	0.3147
**Δ diagnosis-surgery**, years	7.4 ± 2.6	3.1 ± 1.6	0.4437
**Abdominal fistulae**, n (%)	2 (25.0%)	5(62.5%)	0.3147
**Preoperative therapy, n (%)**			
corticosteroids	3 (37.5%)	3 (37.5%)	>0.999
5-ASA	3 (37.5%)	2(25%)	>0.999
**CDAI**	250 (250–300)	300 (250–350)	0.1189
**Type of Surgery**			>0.999
open	1	1	
laparoscopy	7	7	

Data are expressed as mean (±SEM) or median (interquartile range) for continuous variables and numbers (percentages) for categorical variables. IBD = Inflammatory Bowel Disease; CDAI = Clinical Disease Activity Index.

**Table 2:** Post-operative items of the two groups of Crohn’s disease patients.

	Controls	Immunonutrition	*p*
***n***	8	8	
**POD discharge**, days	5 (5–6)	6 (5.2–6.7)	0.2071
**Days of first flatus passage**	4 (2–4)	3 (2–3.75)	0.2890
**Days of first stool**	4(3.25–4.75)	4(3–4.75)	>0.999
**Oral fluid nutrition** n (%)			**0.0256**
POD1	3 (37.5%)	8 (100%)	
POD2–3	5 (62.5%)	0	
**Oral solid nutrition**, n (%)			**0.0286**
POD2	2 (25.0%)	7 (87.5%)	
POD3	2 (25.0%)	1 (12.5%)	
POD4	4 (50.0%)	0	

Data are expressed as median (interquartile range) for continuous variables and numbers (percentages) for categorical variables. Differences between proportions were assessed using the chi-square or Fisher exact test and significant differences were highlighted in bold. POD = postoperative day.

**Table 3:** Mean values of oxidative markers in plasma and/or mucosa samples of Crohn’s disease patients.

Oxidative Markers	Controls	Immunonutrition	*p*
*n*	8	8	
Plasma samples			
**AGEs**, UA/mg of proteins	7.89 ± 1.46	7.56 ± 0.91	0.8560
**AOPPs**, µmol/mg of proteins	1.51 ± 0.38	1.55 ± 0.19	0.9095
Mucosal samples			
**AGEs**, UA/mg of proteins	39.59 ± 4.41	26.83 ± 3.06	**0.0321**
**AOPPs**, µmol/mg of proteins	22.74 ± 6.18	25.15 ± 5.55	0.7753

Data are expressed as means ±SEM. *p* < 0.05 by unpaired *t* test. AOPPs = Advanced Oxidation Protein Products; AGEs = Advanced Glycated End-products; UA = Arbitrary Unit.

## Data Availability

All data supporting the study are available under request.

## References

[1] Casanova M.J., Chaparro M., Molina B., Merino O., Batanero R., Dueñas-Sadornil C., Robledo P., Garcia-Albert A.M., Gómez-Sánchez M.B., Calvet X. (2017). Prevalence of Malnutrition and Nutritional Characteristics of Patients With Inflammatory Bowel Disease. J. Crohn’s Colitis.

[2] Agin M., Yucel A., Gumus M., Yuksekkaya H.A., Tumgor G. (2019). The Effect of Enteral Nutrition Support Rich in TGF-β in the Treatment of Inflammatory Bowel Disease in Childhood. Medicina.

[3] Ruemmele F.M., Roy C.C., Levy E., Seidman E.G. (2000). Nutrition as primary therapy in pediatric Crohn’s disease: Fact or fantasy?. J. Pediatr..

[4] Fell J.M., Paintin M., Arnaud-Battandier F., Beattie R.M., Hollis A., Kitching P., Donnet-Hughes A., MacDonald T.T., Walker-Smith J.A. (2000). Mucosal healing and a fall in mucosal pro-inflammatory cytokine mRNA induced by a specific oral polymeric diet in paediatric Crohn’s disease. Aliment. Pharmacol. Ther..

[5] Grimble R.F. (2005). Immunonutrition. Curr. Opin. Gastroenterol..

[6] Weimann A., Wobith M. ESPEN Guidelines on Clinical nutrition in surgery—Special issues to be revisited. Eur. J. Surg. Oncol..

[7] Wagner I.J., Rombeau J.L. (2011). Nutritional support of surgical patients with inflammatory bowel disease. Surg. Clin. N. Am..

[8] Xu J., Sun X., Xin Q., Cheng Y., Zhan Z., Zhang J., Wu J. (2018). Effect of immunonutrition on colorectal cancer patients undergoing surgery: A meta-analysis. Int. J. Color. Dis..

[9] McClave S.A., Martindale R.G., Maxwell J.P. (2017). Immunonutrition and Colorectal Surgery. Dis. Colon Rectum.

[10] Zhu H., Li Y.R. (2012). Oxidative stress and redox signaling mechanisms of inflammatory bowel disease: Updated experimental and clinical evidence. Exp. Biol. Med..

[11] Christman J.W., Blackwell T.S., Juurlink B.H. (2000). Redox regulation of nuclear factor kappa B: Therapeutic potential for attenuating inflammatory responses. Brain Pathol..

[12] Krzystek-Korpacka M., Neubauer K., Berdowska I., Boehm D., Zielinski B., Petryszyn P., Terlecki G., Paradowski L., Gamian A. (2008). Enhanced formation of advanced oxidation protein products in IBD. Inflamm. Bowel Dis..

[13] Luceri C., Bigagli E., Agostiniani S., Giudici F., Zambonin D., Scaringi S., Ficari F., Lodovici M., Malentacchi C. (2019). Analysis of Oxidative Stress-Related Markers in Crohn’s Disease Patients at Surgery and Correlations with Clinical Findings. Antioxidants.

[14] Ciccocioppo R., Vanoli A., Klersy C., Imbesi V., Boccaccio V., Manca R., Betti E., Cangemi G.C., Strada E., Besio R. (2013). Role of the advanced glycation end products receptor in Crohn’s disease inflammation. World J. Gastroenterol..

[15] Xie F., Sun S., Xu A., Zheng S., Xue M., Wu P., Zeng J.H., Bai L. (2014). Advanced oxidation protein products induce intestine epithelial cell death through a redox-dependent., c-jun N-terminal kinase and poly (ADP-ribose) polymerase-1-mediated pathway. Cell Death Dis..

[16] Shi Y., Qian J., Zhang Q., Hu Y., Sun D., Jiang L. (2020). Advanced glycation end products increased placental vascular permeability of human BeWo cells via RAGE/NF-kB signaling pathway. Eur. J. Obstet. Gynecol. Reprod. Biol..

[17] Kondrup J., Allison S.P., Elia M., Vellas B., Plauth M. (2003). Educational and Clinical Practice Committee, European Society of Parenteral and Enteral Nutrition (ESPEN). ESPEN guidelines for nutrition screening 2002. Clin. Nutr..

[18] Grass F., Pache B., Martin D., Hahnloser D., Demartines N., Hübner M. (2017). Preoperative Nutritional Conditioning of Crohn’s Patients-Systematic Review of Current Evidence and Practice. Nutrients..

[19] Cerantola Y., Grass F., Cristaudi A., Demartines N., Schäfer M., Hübner M. (2011). Perioperative nutrition in abdominal surgery: Recommendations and reality. Gastroenterol. Res. Pract..

[20] Cerantola Y., Hübner M., Grass F., Demartines N., Schäfer M. (2011). Immunonutrition in gastrointestinal surgery. Br. J. Surg..

[21] Moya P., Miranda E., Soriano-Irigaray L., Arroyo A., Aguilar M.D., Bellón M., Muñoz J.L., Candela F., Calpena R. (2016). Perioperative immunonutrition in normo-nourished patients undergoing laparoscopic colorectal resection. Surg. Endosc..

[22] Kellow N.J., Coughlan M.T. (2015). Effect of diet-derived advanced glycation end products on inflammation. Nutr. Rev..

[23] Bigagli E., Luceri C., Dicembrini I., Tatti L., Scavone F., Giovannelli L., Mannucci E., Lodovici M. (2020). Effect of Dipeptidyl-Peptidase 4 Inhibitors on Circulating Oxidative Stress Biomarkers in Patients with Type 2 Diabetes Mellitus. Antioxidants.

[24] Nowotny K., Jung T., Höhn A., Weber D., Grune T. (2015). Advanced glycation end products and oxidative stress in type 2 diabetes mellitus. Biomolecules.

[25] Rojas A., Delgado-López F., González I., Pérez-Castro R., Romero J., Rojas I. (2013). The receptor for advanced glycation end-products: A complex signaling scenario for a promiscuous receptor. Cell. Signal..

[26] Ibrahim Z.A., Armour C.L., Phipps S., Sukkar M.B. (2013). RAGE and TLRs: Relatives., friends or neighbours?. Mol. Immunol..

[27] Body-Malapel M., Djouina M., Waxin C., Langlois A., Gower-Rousseau C., Zerbib P., Schmidt A.M., Desreumaux P., Boulanger E., Vignal C. (2019). The RAGE signaling pathway is involved in intestinal inflammation and represents a promising therapeutic target for Inflammatory Bowel Diseases. Mucosal Immunol..

[28] Snelson M., Lucut E., Coughlan M.T. (2022). The Role of AGE-RAGE Signalling as a Modulator of Gut Permeability in Diabetes. Int. J. Mol. Sci..

[29] Qu W., Yuan X., Zhao J., Zhang Y., Hu J., Wang J., Li J. (2017). Dietary advanced glycation end products modify gut microbial composition and partially increase colon permeability in rats. Mol. Nutr. Food Res..

[30] Sido B., Seel C., Hochlehnert A., Breitkreutz R., Dröge W. (2006). Low intestinal glutamine level and low glutaminase activity in Crohn’s disease: A rational for glutamine supplementation?. Dig. Dis. Sci..

[31] Ji Y., Yang Y., Sun S., Dai Z., Ren F., Wu Z. (2022). Insights into diet-associated oxidative pathomechanisms in inflammatory bowel disease and protective effects of functional amino acids. Nutr. Rev..

[32] Crespo I., San-Miguel B., Prause C., Marroni N., Cuevas M.J., González-Gallego J., Tuñón M.J. (2012). Glutamine treatment attenuates endoplasmic reticulum stress and apoptosis in TNBS-induced colitis. PLoS ONE.

[33] Wang H., Zhang C., Wu G., Sun Y., Wang B., He B., Dai Z., Wu Z. (2015). Glutamine enhances tight junction protein expression and modulates corticotropin-releasing factor signaling in the jejunum of weanling piglets. J. Nutr..

[34] Wang B., Wu Z., Ji Y., Sun K., Dai Z., Wu G. (2016). L-Glutamine Enhances Tight Junction Integrity by Activating CaMK Kinase 2-AMP-Activated Protein Kinase Signaling in Intestinal Porcine Epithelial Cells. J. Nutr..

[35] Singh K., Gobert A.P., Coburn L.A., Barry D.P., Allaman M., Asim M., Luis P.B., Schneider C., Milne G.L., Boone H.H. (2019). Dietary Arginine Regulates Severity of Experimental Colitis and Affects the Colonic Microbiome. Front. Cell. Infect. Microbiol..

[36] Rao J.N., Liu L., Zou T., Marasa B.S., Boneva D., Wang S.R., Malone D.L., Turner D.J., Wang J.Y. (2007). Polyamines are required for phospholipase C-gamma1 expression promoting intestinal epithelial restitution after wounding. Am. J. Physiol. Gastrointest. Liver Physiol..

[37] Lecleire S., Hassan A., Marion-Letellier R., Antonietti M., Savoye G., Bôle-Feysot C., Lerebours E., Ducrotté P., Déchelotte P., Coëffier M. (2008). Combined glutamine and arginine decrease proinflammatory cytokine production by biopsies from Crohn’s patients in association with changes in nuclear factor-kappaB and p38 mitogen-activated protein kinase pathways. J. Nutr..

[38] Drover J.W., Dhaliwal R., Weitzel L., Wischmeyer P.E., Ochoa J.B., Heyland D.K. (2011). Perioperative use of arginine-supplemented diets: A systematic review of the evidence. J. Am. Coll. Surg..

[39] Lev-Tzion R., Griffiths A.M., Leder O., Turner D. (2014). Omega 3 fatty acids (fish oil) for maintenance of remission in Crohn’s disease. Cochrane Database Syst. Rev..

[40] Bischoff S.C., Forbes A., Escher J., Hébuterne X., Kłęk S., Krznaric Z., Schneider S., Shamir R., Stardelova K., Wierdsma N. (2017). ESPEN guideline: Clinical nutrition in inflammatory bowel disease. Clin. Nutr..

[41] Barbalho S.M., Goulart Rde A., Quesada K., Bechara M.D., de Carvalho Ade C. (2016). Inflammatory bowel disease: Can omega-3 fatty acids really help?. Ann. Gastroenterol..

[42] Beguin P., Errachid A., Larondelle Y., Schneider Y.J. (2013). Effect of polyunsaturated fatty acids on tight junctions in a model of the human intestinal epithelium under normal and inflammatory conditions. Food Funct..

[43] Zhao J., Shi P., Sun Y., Sun J., Dong J.N., Wang H.G., Zuo L.G., Gong J.F., Li Y., Gu L.L. (2015). DHA protects against experimental colitis in IL-10-deficient mice associated with the modulation of intestinal epithelial barrier function. Br. J. Nutr..

[44] Tanaka M., Lee K., Martinez-Augustin O., He Y., Sanderson I.R., Walker W.A. (1996). Exogenous nucleotides alter the proliferation, differentiation and apoptosis of human small intestinal epithelium. J. Nutr..

[45] Hess J.R., Greenberg N.A. (2012). The role of nucleotides in the immune and gastrointestinal systems: Potential clinical applications. Nutr. Clin. Pract..

[46] Heerasing N., Thompson B., Hendy P., Heap G.A., Walker G., Bethune R., Mansfield S., Calvert C., Kennedy N.A., Ahmad T. (2017). Exclusive enteral nutrition provides an effective bridge to safer interval elective surgery for adults with Crohn’s disease. Aliment. Pharmacol. Ther..

[47] Beaupel N., Brouquet A., Abdalla S., Carbonnel F., Penna C., Benoist S. (2017). Preoperative oral polymeric diet enriched with transforming growth factor-beta 2 (Modulen) could decrease postoperative morbidity after surgery for complicated ileocolonic Crohn’s disease. Scand. J. Gastroenterol..

[48] Waitzberg D.L., Saito H., Plank L.D., Jamieson G.G., Jagannath P., Hwang T.L., Mijares J.M., Bihari D. (2006). Postsurgical infections are reduced with specialized nutrition support. World J. Surg..

